# Stable cerebrospinal fluid neurogranin and β-site amyloid precursor protein cleaving enzyme 1 levels differentiate predementia Alzheimer’s disease patients

**DOI:** 10.1093/braincomms/fcac244

**Published:** 2022-09-24

**Authors:** Bjørn Eivind Kirsebom, Grit Richter, Kaja Nordengen, Dag Aarsland, Geir Bråthen, Betty M Tijms, Pieter Jelle Visser, Johanna Nilsson, Per Selnes, Milica G Kramberger, Bengt Winblad, Knut Waterloo, Berglind Gísladóttir, Kaj Blennow, Tormod Fladby

**Affiliations:** Department of Neurology, University Hospital of North Norway, 9038 Tromsø, Norway; Department of Psychology, Faculty of Health Sciences, UiT, The Arctic University of Norway, 9019 Tromsø, Norway; Department of Neurology, University Hospital of North Norway, 9038 Tromsø, Norway; Department of clinical medicine, Faculty of Health Sciences, UiT The Arctic University of Norway, 9019 Tromsø, Norway; Department of Neurology, Akershus University Hospital, 1478 Lørenskog, Norway; Centre for Age-Related Medicine, Stavanger University Hospital, 4068 Stavanger, Norway; Department of Old Age Psychiatry, Institute of Psychiatry, Psychology and Neuroscience, King’s College London, London SES 8AF, UK; Department of Neuromedicine and Movement Science, Faculty of Medicine and Health Sciences, Norwegian University of Science and Technology, 7491 Trondheim, Norway; Department of Neurology and Clinical Neurophysiology, University Hospital of Trondheim, 7030 Trondheim, Norway; Alzheimer Center Amsterdam, Department of Neurology, Amsterdam Neuroscience, Vrije Universiteit Amsterdam, Amsterdam UMC - Location VUmc, 1007 MB Amsterdam, The Netherlands; Alzheimer Center Amsterdam, Department of Neurology, Amsterdam Neuroscience, Vrije Universiteit Amsterdam, Amsterdam UMC - Location VUmc, 1007 MB Amsterdam, The Netherlands; Alzheimer Center Limburg, School for Mental Health and Neuroscience, Maastricht University, 6200 MD Maastricht, The Netherlands; Department of Neurobiology, Care Sciences and Society, Division of Neurogeriatrics, Karolinska Institutet, 171 77 Stockholm, Sweden; Department of Psychiatry and Neurochemistry, Institute of Neuroscience and Physiology, The Sahlgrenska Academy, University of Gothenburg, 413 45 Gothenburg, Sweden; Department of Neurology, Akershus University Hospital, 1478 Lørenskog, Norway; Centre for cognitive impairments, Department of Neurology, University Medical Centre, 1000 Ljubljana, Slovenia; Department of Neurology, Medical faculty, University of Ljubljana, 1000 Ljubljana, Slovenia; Department of Neurobiology, Care Sciences and Society, Division of Neurogeriatrics, Karolinska Institutet, 171 77 Stockholm, Sweden; Karolinska University Hospital, Theme Inflammation and Aging, 141 86 Stockholm, Sweden; Department of Neurology, University Hospital of North Norway, 9038 Tromsø, Norway; Department of Psychology, Faculty of Health Sciences, UiT, The Arctic University of Norway, 9019 Tromsø, Norway; Department of Neurology, Akershus University Hospital, 1478 Lørenskog, Norway; Clinical Molecular Biology (EpiGen), Medical Division, University of Oslo, 1478 Ahus, Lørenskog, Norway; Department of Psychiatry and Neurochemistry, Institute of Neuroscience and Physiology, The Sahlgrenska Academy, University of Gothenburg, 413 45 Gothenburg, Sweden; Clinical Neurochemistry Laboratory, Sahlgrenska University Hospital, 431 30 Mölndal, Sweden; Department of Neurology, Akershus University Hospital, 1478 Lørenskog, Norway; Institute of Clinical Medicine, University of Oslo, Campus Ahus, 1478 Lørenskog, Norway

**Keywords:** Alzheimer’s disease, synaptic loss, neurogranin, BACE1, A/T/N

## Abstract

Cerebrospinal fluid (CSF) β-site amyloid precursor protein cleaving enzyme 1 (BACE1), neurogranin and the neurogranin/BACE1 ratio are proposed markers for Alzheimer’s disease. BACE1 is also a drug target. However, CSF levels may differ between early-stage amyloid plaque formation (A) and later stage downstream tau-tangle pathology (T) and neurodegeneration (N) and may be expressed as an A/T/N stage (e.g. A+/T−/N or A+/T+/N+). Whether BACE1 and neurogranin levels are persistent traits or change with disease progression is unknown. The aim of this study was to investigate whether CSF neurogranin and BACE1 concentrations differ between A/T/N stages, whether these change over time and correlate with memory decline. This may have implications for patient selection in future trials. We used CSF markers to determine A/T/N stage using amyloid beta42/40 ratio, p-tau181 and total-tau respectively in predementia Alzheimer’s disease cases (*n* = 176) [including cases that progressed to dementia (*n* = 10)] and controls (*n* = 74) from the Norwegian Dementia Disease Initiation cohort. We selected cases at the presumed early (A+/T−/N−, *n* = 86) and late stages (A+/T+/N+, *n* = 90) of the Alzheimer’s disease continuum and controlled with normal markers (A−/T−/N−, *n* = 74). A subset of subjects in all A/T/N groups underwent repeat CSF sampling at approximately 2-year intervals up to 6 years from baseline. Using linear mixed models, longitudinal measurements of CSF BACE1 and neurogranin levels in A+/T−/N− and A+/T+/N+ as compared to A−/T−/N− healthy controls were performed. Next, we measured changes in CSF BACE1 and neurogranin levels in cases that progressed from A−/T−/N− to A+/T−/N− (*n* = 12), from A+/T−/N− to A+/T or N+ (*n* = 12), remained stable A+/T−/N− (*n* = 26), remained stable A+/T+/N+ (*n* = 28) compared with controls remaining stable A−/T−/N− (*n* = 33). Lastly, associations between these markers and memory decline were assessed. Compared with A−/T−/N− healthy controls, neurogranin was unaltered in A+/T−/N− (n.s.) but higher in A+/T+/N+ (*P* < 0.0001). In contrast, BACE1 was lower in A+/T−/N− (*P* < 0.05) and higher in A+/T+/N+ (*P* < 0.0001). The neurogranin/BACE1 ratio was increased in both A+/T−/N− (*P* < 0.05) and A+/T+/N+ (*P* < 0.0001) groups as compared to A-/T-/N- healthy controls and was more strongly associated with memory decline (b = −0.29, *P* = 0.0006) than neurogranin (b = −0.20, *P* = 0.002) and BACE1 (b = −0.13, *P* = 0.046). Neurogranin and BACE1 level differences remained stable over time not only within A/T/N groups but also in patients progressing to more pathological A/T/N stages (e.g. progressing from A+/T−/N− to A + T or N+) and in cases progressing to dementia. Our results suggest that neurogranin and BACE1 levels may differentiate pathomechanistic Alzheimer’s disease subgroups, putatively with different options for treatment.

## Teaser

Kirsebom & Richter et al. report that levels of the CSF synapse markers neurogranin and BACE1 remain stable in Alzheimer’s disease A/T/N subgroups, even when progressing to dementia. Their results suggest that CSF levels may differentiate pathomechanistic Alzheimer’s disease subtypes, putatively with different options for treatment.

## Graphical Abstract

**Graphical abstract:**
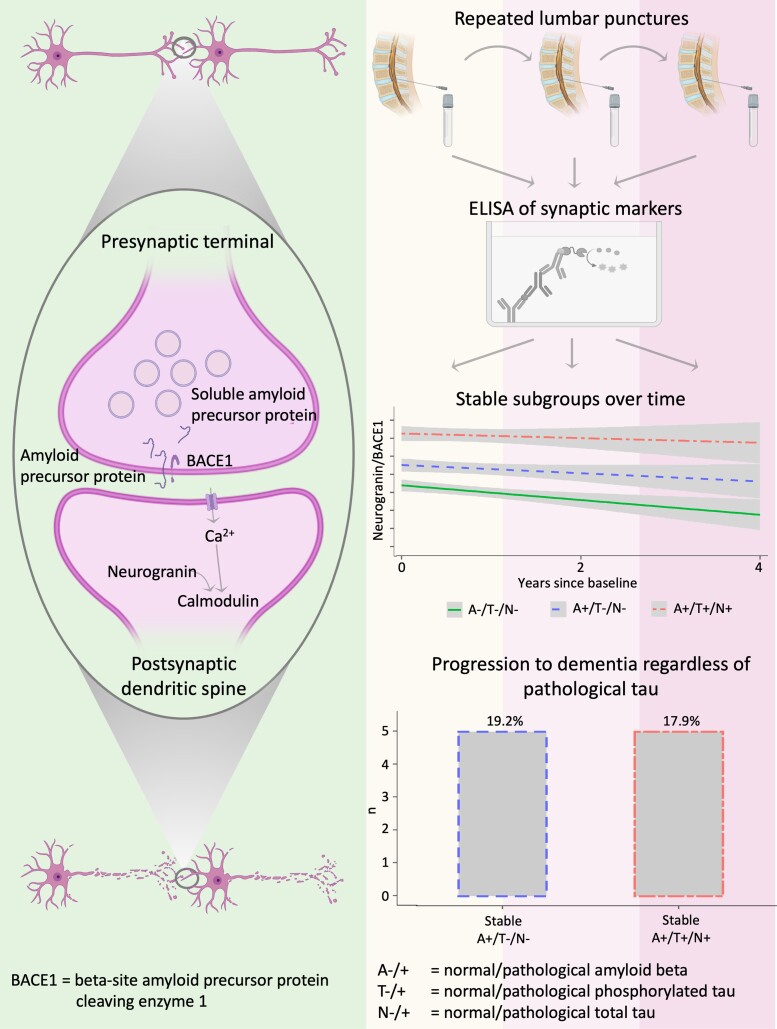

## Introduction

According to the amyloid cascade hypothesis,^1^ the Alzheimer’s disease-continuum is initiated by amyloid-beta (Aβ) dysmetabolism and formation of amyloid plaques (A), followed by the emergence of tau-tangle pathology (T) and neurodegeneration (N) commonly expressed within the A/T/N system.^2^ Additional pathologies are also well documented in Alzheimer’s disease, and synapse degeneration contributes to symptoms and may contribute to the progression of core pathologies. However, Alzheimer’s disease pathology is heterogeneous and differentially expressed between patients, both in terms of core pathologies and synapse degeneration.^3^ These differences may result from examination at different stages of disease progression or the existence of consistent Alzheimer’s disease subtypes or endophenotypes, potentially in need of different treatment approaches.^2,4,5^

Generation of potentially harmful Aß-species from amyloid precursor protein (APP) is linked to presynaptic beta-site APP cleaving enzyme 1 (BACE1) activity and is associated with accumulation of Aβ oligomers and loss of pre-synaptic machinery, which is pronounced in Alzheimer’s disease.^6^ Aβ has presynaptic effects on receptors and synaptic transmission and oligomers may upregulate BACE1 expression.^7,8^ Post-synaptic effects of Aβ oligomers are thought to occur by overstimulation of N-methyl-D-aspartate (NMDA) receptors with related Ca^2+^ influx and postsynaptic hyperexcitation.^9–12^ Neurogranin (Ng) and BACE1 have been proposed as biomarkers for Alzheimer’s disease pathophysiology and progression. Increased CSF levels of the postsynaptic protein Ng are a marker of synaptic dysfunction and predict clinical progression in Alzheimer’s disease,^13–15^ though increased levels are not specific to Alzheimer’s disease dementia.^16^

Ng is abundant in the hippocampus, particularly in pyramidal neurones in the CA3 subfield and is required for synaptic plasticity, long-term potentiation and memory consolidation, likely through involvement in spine Ca^2+^/calmodulin signal transduction.^17–20^ BACE1 is expressed in presynaptic terminals, abundantly in the hippocampus and particularly in mossy fibre terminals in CA3 pyramidal cells.^7,21,22^ Experimental data suggest that these synapses are susceptible to early (pre-plaque) loss of plasticity in Alzheimer’s disease.^23^ BACE1 has a multitude of roles in proteolysis and synaptic functioning.^7,11^ Importantly, BACE1 proteolysis is rate-limiting for Aβ formation, and BACE1-site APP-mutations are linked to familial Alzheimer’s disease. BIN1 and EphA4 are genetic risk factors for Alzheimer’s disease that mediate Aβ production via regulation of BACE1 transport and activity.^24,25^ These findings motivated the development and testing of BACE1 inhibitors in drug trials, without reaching treatment endpoints and with indications of negative cognitive effects, other adverse effects, including regionally increased brain atrophy.^26–29^ Side effects of BACE1 inhibitors are both on-target and off-target, and specificity for BACE1 inhibitors tested in clinical trials is also limited.^30^ However, BACE1 is still an attractive target in Alzheimer’s disease and the possibility that novel alternatives for BACE1 inhibition may improve treatment specificity, and efficacy in biomarker-defined subgroups of patients cannot be excluded.^7,11^

The mossy fibre CA3 synapses contain NMDA types of glutamate receptors with different subunits expressed at post- and pre-synaptic (recurrent connections) locations.^31^ While the pre-synaptic receptors may have a modulatory role, excitotoxicity may be induced at post-synaptic or extra-synaptic receptors, where Aß-oligomers may induce spine loss and neurodegeneration, leading to Ng release in Alzheimer’s disease.^12,32–34^ Memantine is currently the only clinically approved NMDA-receptor antagonist used in Alzheimer’s disease. It is a low-affinity NMDA-receptor blocker currently used mainly for symptomatic treatment,^35^ but efficacy towards disease progression has also been suggested (i.e. preserved hippocampal volumes and reduced CSF p-tau levels).^36,37^ It acts preferentially at extra-synaptic receptors and allows physiological signalling. However, a combination of receptor subunit-specific selective agonists and antagonists may be beneficial, and several promising candidate drugs are in the pipeline.^12^ If treatment can be focused on particularly susceptible subgroups, drug effects could emerge.^12,34^

We and others have previously shown that the ratio between Ng and BACE1 (Ng/BACE1) is increased in subjective cognitive decline (SCD) and mild cognitive impairment (MCI) with amyloid plaques, and is more strongly related to hippocampal volume, cognitive impairment and decline as compared to Ng alone.^17,38^ However, Ng and BACE1 levels in patients with only amyloid plaque pathology (A+/T−/N−) has not been compared to more advanced stages (i.e. A+/T+/N+). Moreover, longitudinal studies of these biomarkers are sparse, and it is unknown if differences between cases persist, or if levels are altered with accumulating Alzheimer’s disease pathology. The aim of this study is to investigate longitudinal differences in Ng, BACE1 and Ng/BACE1 cerebrospinal fluid (CSF) levels in predementia Alzheimer’s disease patients as a prequel to drug trials stratified for putative subgroups with increased susceptibility to associated Alzheimer’s disease mechanisms. If Ng and BACE1 levels are consistent traits, they may point to Alzheimer’s disease subtypes and the need for different treatment strategies. Alternatively, transitions in Ng and BACE1 levels may follow plaque deposition (transition to A+), acquisition of significant amounts of neurofibrillary tangle pathology (T+) or neurodegeneration (N+) as measured by pertinent CSF markers. In addition, we explore longitudinal associations with memory impairment.

## Methods and materials

### Study population

This study was a part of the Norwegian multi-centre study, Dementia disease initiation (DDI). The DDI cohort consists of non-demented individuals between 40 and 80 years of age, primarily recruited from memory clinics and advertisements in local news media. For a detailed description of inclusion and exclusion criteria, please see Fladby *et al.* (2017).^39^ We included participants with pathological levels of CSF Aβ_42/40_ ratio and/or pathological levels of CSF phosphorylated tau181 (p-tau) and total tau (t-tau) who were originally recruited as healthy controls (*n* = 22) or classified either as SCD (*n* = 57) or MCI (*n* = 97), as well as healthy controls with normal levels of CSF Aβ_42/40_ ratio, p-tau and t-tau (*n* = 74). Participants were classified as SCD according to the SCD-I framework, which requires normal performance on neuropsychological tests while experiencing a subjective decline in any cognitive domain.^40^ MCI was classified according to the NIA-AA criteria, which require the presence of subjective cognitive impairment or decline in combination with lower performance than expected in one or more cognitive domains, yet preserved independence in functional ability and not fulfilling the criteria of dementia.^41^ Healthy controls reported no SCD and were recruited from spouses of patients with dementia/cognitive disorder and patients who completed lumbar puncture for orthopaedic surgery. Cognitive impairment was determined when results were 1.5 standard deviation below the normative mean within one or more cognitive domains, including delayed memory recall [Consortium to Establish a Registry for Alzheimer’s Disease (CERAD) word list test],^42,43^ executive function (Trail Making Test part B),^44,45^ language/verbal fluency (Controlled Oral Word Association Test)^45,46^ and visuoperceptual ability (Visual Object and Space Perception Battery (VOSP) silhouettes).^47^

### CSF collection, storage and analysis and genetics

Lumbar punctures were performed following a detailed BIOMARKAPD SOP as described previously.^48^ Briefly, sampling was done between 9 and 12 AM noon, and CSF was collected in polypropylene tubes (Thermo Fisher Scientific, MA, USA) which were centrifuged within 2 h at 2000 g for 10 min at room temperature. The supernatant was then transferred to new defined tubes, directly frozen at − 80 °C and kept at − 80 °C until thawed for analysis.

The QuickPlex SQ120 system from MesoScale discovery (MSD, MD, USA) was used to measure Aβ_1-42_ and Aβ_1-40_ in a multiplex setup using the V-plex Ab Peptide Panel 1 (6E10) kit (K15200E-1). The analyses were carried out according to the manufacturers’ procedures. Commercial enzyme-linked immunosorbent assays (Innotest, Fujirebio, Ghent, Belgium) based on monoclonal antibodies were used to measure CSF concentrations of total tau (t-tau) using hTau Ag kits and phosphorylated tau (p-tau) using 181P kits. BACE1 and Ng (trunc P75) concentrations were determined using kits provided by EUROIMMUN AG (Lübeck, Germany), as described in detail elsewhere.^17^ All samples were analyzed in duplicates and reanalyzed if relative deviations (RDs) exceeded 20% and quality control samples with an RD threshold of 15% were controlled for interplate and interday variation*. APOE* genotyping was performed on EDTA blood samples as previously described.^39^

### A/T/N classification and study design

The A/T/N classification scheme^2^ for biomarkers of hallmark Alzheimer’s disease pathology was used to determine the presence of amyloid plaques (A), neurofibrillary tangles (T) and evidence of neurodegeneration (N) from CSF Aβ_42/40_ ratio, p-tau and t-tau, respectively. The following cut-off values for CSF t-tau and p-tau abnormalities were applied according to the laboratory recommendations (modified from the study by Sjogren *et al.* 2001^49^): t-tau ≥300 pg/mL for age ≤50 years, ≥450 pg/mL for ages 50 to 69 years and ≥500 pg/mL for ages ≥70 years, and p-tau ≥80 pg/mL. An optimum cut-off for Aβ_42/40_ ratio at ≤0.077 was determined following receiver operating curve (ROC) analysis using visual read of [18F]-Flutemetamol PET scans as the standard of truth.^50^ We selected three groups based on the A/T/N staging at baseline: (i) cases with amyloid pathology without tau pathology (A+/T−/N−, *n* = 86), (ii) cases with both amyloid pathology and tau pathology (A+/T+/N+, *n* = 90), and (iii) healthy controls with normal CSF Alzheimer’s disease biomarkers (A−/T−/N−, *n* = 74). Longitudinally collected CSF samples were available for subsets from all groups (A-/T-/N-: *n* = 33; A+/T-/N-: *n* = 39; A+/T+/N+: *n* = 28) at approximately 2-year intervals ranging between one and 6 years from baseline (see Supplementary Table 1 for details). In a sub-analysis, we included (i) healthy controls with A−/T−/N− staging at both baseline and at least one subsequent follow-up visit (*n* = 33), (ii) cases that remained stable A+/T−/N− over time (*n* = 26), (iii) all cases that progressed from A+/T−/N− to A+/T or N+ (A+/T/N+, *n* = 12), (iv) stable A+/T+/N+ cases (*n* = 28), and (v) we additionally included all cases that progressed from A−/T−/N− to A+/T−/N− staging from the DDI database (new cases not included in the main analysis, *n* = 12). Details are summarized in Supplementary Table 2.

### Cognitive tests

Due to previous findings showing that CSF Ng and Ng/BACE1 were primarily associated with memory performance,^38^ the CERAD word list delayed memory recall subtest^42^ was selected for associations with synapse markers in the present study.

### Ethics

The participants signed written informed consent and the study was approved by the Regional Ethics board (REK 2013/150). The study conducted was in line with the guidelines provided by the Helsinki declaration of 1964 (revised 2013) and the Norwegian Health and Research Act.

### Statistical analyses

Statistical analyses were performed with R version 4.0.2.^51^ A/T/N group differences for age and education at baseline were measured with one-way ANOVA. For CERAD delayed memory, ANCOVA with age, education and sex as covariates was used. Chi-square tests were used for sex, diagnosis (healthy control, SCD and MCI) and *APOE-ε*4 carrier status between groups. ANCOVA with age and *APOE-ε*4 carrier status as covariates was used to measure group differences in CSF synapse marker levels. To reduce the family-wise error rate associated with multiple testing of the same markers individually and as a ratio, the Holm-Bonferroni sequential procedure was used for post-hoc comparisons. Linear mixed models (LMMs) were fitted to assess the longitudinal trajectories of CSF Ng, BACE1 and Ng/BACE1 ratio, Aβ_42/40_ ratio, p-tau and t-tau in the different A/T/N groups with age and *APOE-ε*4 carrier status included as covariates. We used LMMs to assess the relationship between baseline levels of CSF synapse markers and future CERAD memory decline with age, years of education and sex included as covariates.^43^ For both baseline and longitudinal models, all CSF markers except the Ng/BACE1 and Aβ_42/40_ ratios were log-transformed, and all continuous variables were standardized (z-values) prior to analyses. For cognitive analyses, we determined that the inclusion of a random intercept for cognitive status at baseline improved model fit (determined by the Bayesian Information Criterion). Cognitive status was operationalized as demographically adjusted normative CERAD recall scores^43^ that were greater than or less than 1.5 standard deviations from the mean. As Ng and BACE1 were measured both individually and as a ratio and because most of the cases were measured twice in the longitudinal main- and sub-analyses of the A/T/N groups, we opted for a more stringent α-level (0.01) in the longitudinal models. Following the findings from our analyses, we performed two supplementary analyses. (i) ANCOVA between baseline A/T/N groups for Aβ_1-42,_ Aβ_1-40_ and the Aβ_42/40 ratio_ with age and *APOE-ε*4 carrier status as covariates (post-hoc Holm-Bonferroni), (ii) Pearson’s correlations between baseline values of Ng and BACE1 in A/T/N groups that progressed to more pathological A/T/N stages or remained stable and (iii) a descriptive comparison of progression to dementia between A/T/N groups.

### Data availability

Data from the DDI cohort are stored at Services for sensitive data (TSD) at the University of Oslo and are publicly unavailable. However, anonymized data used in this study may be made available by the corresponding author upon reasonable request.

## Results

### Baseline A/T/N group differences of CSF Ng, BACE1, and Ng/BACE1 levels

**Figure 1:**
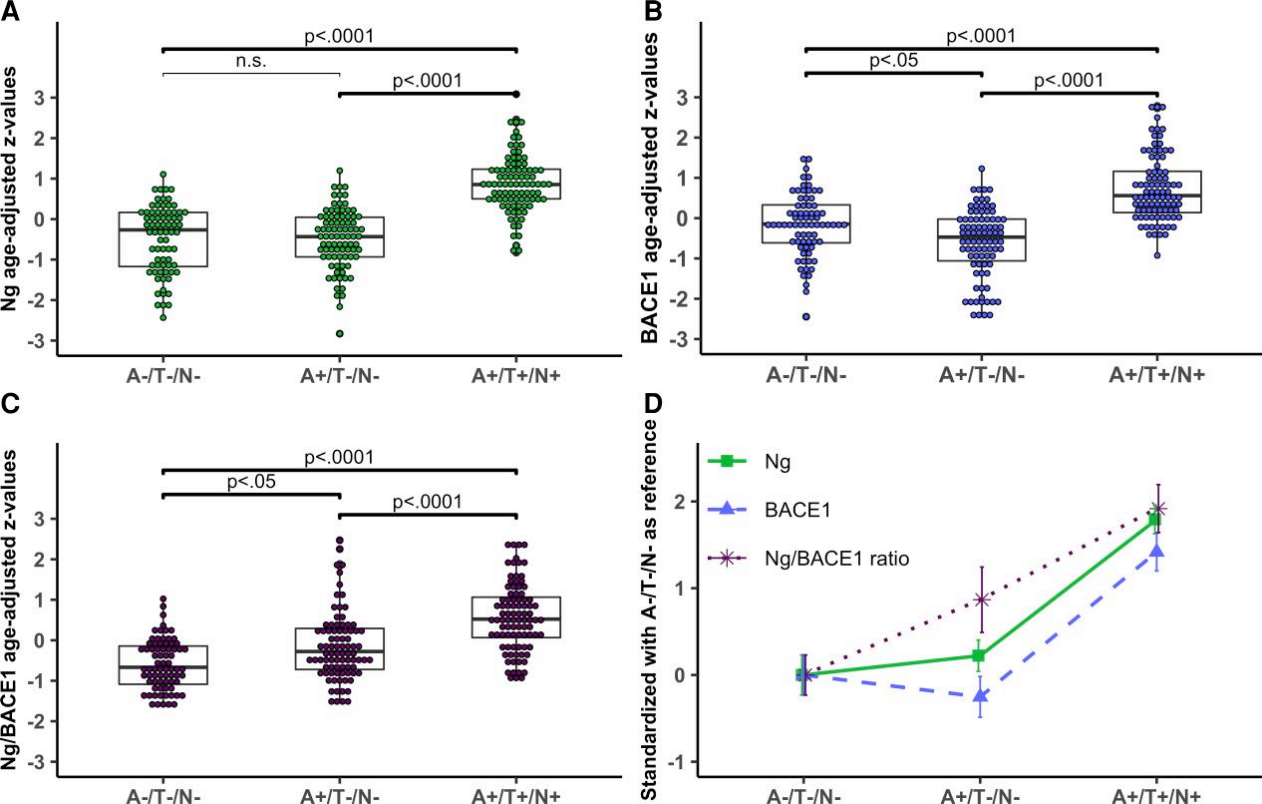
**Baseline BACE1, Ng and Ng/BACE1 ratio between A/T/N groups.** (**A–C**) The z-values (y-axis) are age-adjusted regression residuals. The brackets mark the statistical comparisons of interest following a significant ANCOVA main-effect between the A/T/N groups. The *P*-values are in accordance with the threshold following the Bonferroni-Holm procedure. (**D**) The z-values (y-axis) are standardized z-values created with the A-T-N- healthy control group as a reference. The error bars display standard deviations.

CSF Ng levels were similar to A−/T−/N− controls in A+/T−/N− cases (n.s.) but higher in A+/T+/N+ (*P* < 0.0001). (Table 1, Figs. 1A & D). For BACE1, levels were lower in A+/T−/N− cases compared with A−/T−/N− controls (*P* < 0.05) and higher in A+/T+/N+ cases (*P* < 0.0001) and thus higher in A+/T+/N+ compared to A+/T−/N− cases (*P* < 0.0001) (Table 1, Figs. 1B and D). Ng/BACE1 levels were higher in both A+/T−/N− (*P* < 0.05) and A+/T+/N+ cases (*P* < 0.0001) compared with A−/T−/N− controls, and Ng/BACE1 was higher in A+/T+/N+ compared to A+/T−/N− (*P* < 0.0001) (Table 1 and Figs. 1C and D).

**Table 1:** Between-group comparisons of baseline cognitive characteristics and CSF synapse markers

	A/T/N groups (n)			*F*/*χ^2^*/*η^2^*/*η_p_^2^ (P)*	Statistical tests		
	A−/T−/N− (74)	A+/T−/N− (86)	A+/T+/N+ (90)		A−/T−/N− *versus* A+/T−/N−	A−/T−/N− *versus* A+/T+/N+	A+/T−/N− *versus* A+/T+/N+
**Age** mean (SD)	60.9 (8.3)	67.9 (7.4)	68.0 (8.4)	*F* = 20.9, *η^2^*=0.15 (**<0.0001**)	^a^ **<** 0**.0001**	^a^ **<** 0**.0001**	^a^n.s.
**Years of education** mean (SD)	14.3 (3.2)	14.0 (3.4)	13.1 (3.1)	*F* = 3.1, *η^2^*=0.03 (**<0.05**)	^b^n.s.	^b^ **<** 0**.05**	^b^n.s.
**Female** **n (%)**	44 (59.5)	52 (60.5)	46 (51.1)	*χ^2^* = 1.9, (n.s.)	^c^	^c^	^c^
**CERAD recall** mean (SD)	7.64 (1.9) *n* = 73	5.4 (2.7) *n* = 85	3.6 (2.9) *n* = 89	*F* = 35.6, *η_p_^2^*=0.23 (**<0.0001**)	^d^ **<** 0**.0001**	^d^ **<** 0**.0001**	^d^ **<** 0**.0001**
**Recruited as Controls**	74 (77.1)	15 (15.6)	7 (7.3)	χ^2^ = 83.7 (**<0.0001**)	^c^	^c^	^c^
**SCD**	†	33 (58.9)	23 (41.1)	χ^2^ = 1.8 (n.s.)	^c^	^c^	^c^
**MCI**	†	36 (37.5)	60 (62.5)	χ^2^ = 6.0 (**<0.05**)	^c^	^c^	^c^
***APOE-ε4-*** *n* (%)	46 (53.5)	21 (24.4)	19 (22.1)	χ^2^ = 15.8 (**<0.0001**)	^c^	^c^	^c^
***APOE-ε4+*** *n* (%)	28 (17.1)	65 (39.6)	71 (43.3)	χ^2^ = 19.8 (**<0.0001**)	^c^	^c^	^c^
**Ng** mean (SD)	295.4(316.4)	316.36 (101.6)	594.8 (205.7)	*F* = 87.0, *η_p_^2^*=0.42 (**<0.0001**)	^d^n.s.	^d^ **<** 0**.0001**	^d^ **<** 0**.0001**
**BACE1** mean (SD)	2091.9 (536.7)	1967.4 (522.2)	2396.6 (850.5)	*F* = 61.1, *η_p_^2^*=0.33 (**<0.0001**)	^d^ **<** 0**.05**	^d^ **<** 0**.0001**	^d^ **<** 0**.0001**
**Ng/BACE1** mean (SD)	0.139 (0.03)	0.165 (0.05)	0.196 (0.04)	*F* = 28.5, *η_p_^2^* = 0.19 (**<0.0001**)	^d^ **<** 0**.05**	^d^ **<** 0**.0001**	^d^ **<** 0**.0001**

A+/**−**, positive or negative CSF marker for amyloid plaques; N+/**−**, positive or negative marker for neurodegeneration; SD, standard deviation; n, number of cases; %, percentage; F, F statistic; χ2, chi square statistic; η2, eta-squared; ηp2, partial eta-squared. ^a^no value; ^b^ANOVA post-hoc; ^c^no post-hoc comparisons performed; ^d^ANCOVA comparisons (Bonferroni-Holm). Statistically significant results are highlighted in bold.

### Longitudinal trajectories of CSF markers for baseline A/T/N groups

**Figure 2:**
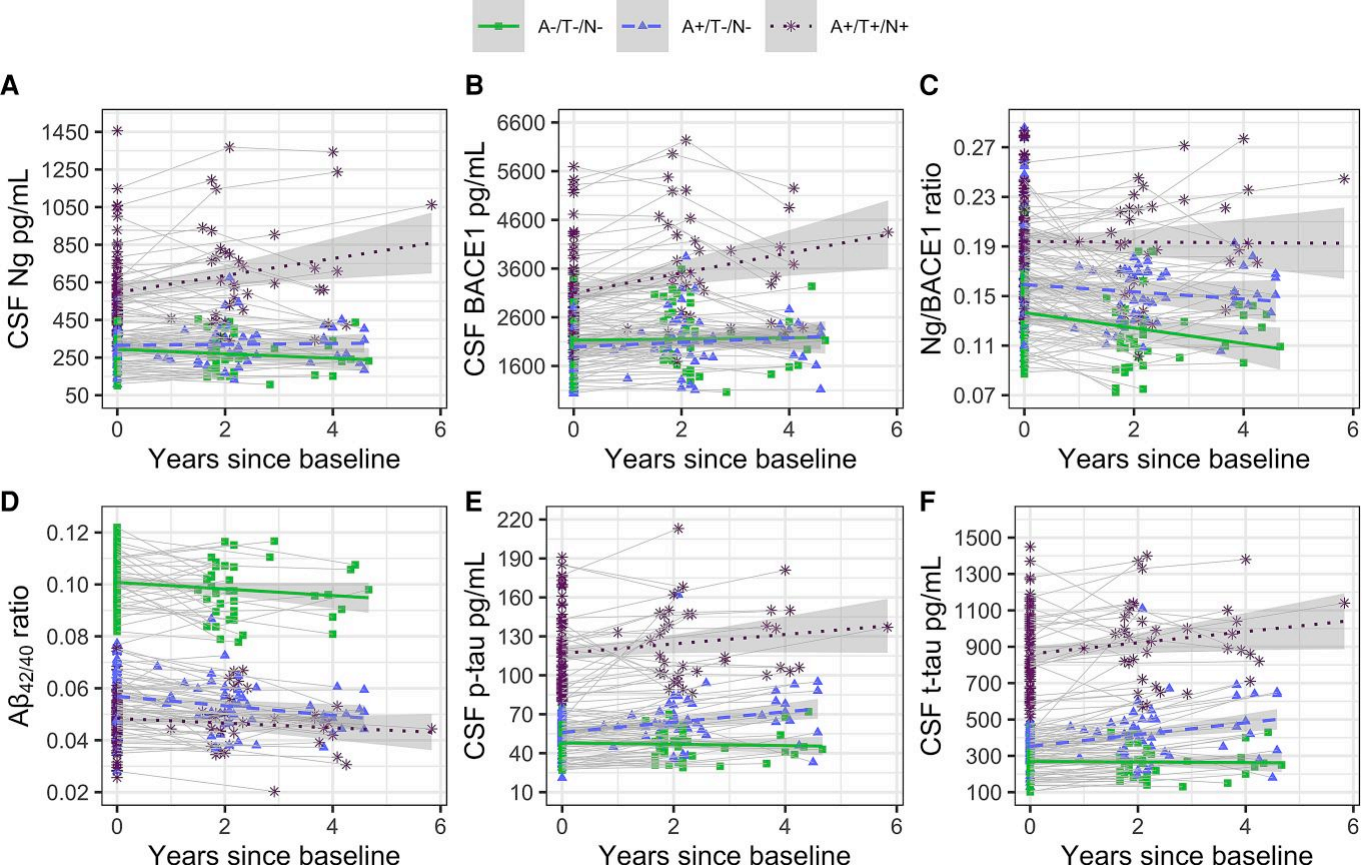
**Longitudinal change within and between baseline-determined A/T/N groups using LMMs. A–C** show longitudinal changes in Ng, BACE1 and Ng/BACE1 ratios. **D–F** show longitudinal changes in Aß_42/40_ ratios, p-tau and t-tau. All compared to stable A−/T−/N− controls: Baseline Ng only higher in A+/T+/N+ (*P* < 0.0001), BACE1 lower in A+/T−/N− (*P* = 0.006) and higher in A+/T+/N+ (*P* < 0.0001). Ng/BACE1 is higher in both A+/T−/N− (*P* = 0.0003) and A+/T+/N+ (*P* < 0.0001). Ng, BACE1 and Ng/BACE1 levels and Aß_42/40_ ratios remained stable over time in respective A/T/N groups (all n.s. change over time). Higher baseline p-tau (*P* = 0.001) and t-tau (*P* < 0.0001) levels in A+/T−/N− compared to A−/T−/N− controls and increases in both tau markers (*P* = 0.01; *P* = 0.004) over time.

Please see Table 2 and Fig. 2 for details. No significant ATN group-by-time interactions were demonstrated for BACE1, Ng, or Ng/BACE1 ratio, suggesting that levels for synapse markers remain largely unchanged within A/T/N groups over time. In contrast, while the Aβ_42/40_ ratio remained stable in all groups, the A+/T−/N− group had higher p-tau (*P* = 0.001) and t-tau (*P* < 0.0001) levels than A−/T−/N− controls and also showed significant increases in both tau markers (*P* = 0.01; *P* = 0.004) over time.

**Table 2:** Longitudinal mixed linear models of CSF synapse marker change by A/T/N group

	Predictors	b	SE	95% CI	t	*P*
**CSF BACE1**	Intercept	−0.17	0.10	−0.36 – 0.02	−1.71	0.087
	Age	0.22	0.05	0.11–0.32	3.96	**<0**.**0001**
	APOE4 status	−0.05	0.11	−0.27 – 0.16	−0.49	0.624
	Time	0.05	0.04	−0.02 – 0.13	1.38	0.168
	A+/T−/N−	−0.38	0.14	−0.64 – −0.11	−2.74	0.**006**
	A+/T+/N+	0.86	0.14	0.59–1.13	6.22	**<0**.**0001**
	A+/T−/N−*Time	-0.05	0.05	−0.15 – 0.04	−1.13	0.259
	A+/T+/N+*Time	-0.06	0.05	−0.16 – 0.04	−1.23	0.217
	*Predictors*	*b*	*SE*	*95% CI*	*t*	*P*
**CSF Ng**	Intercept	−0.56	0.09	−0.74 – −0.38	−6.17	**<0**.**0001**
	Age	0.11	0.05	0.01–0.21	2.19	**<0**.**05**
	APOE4 status	0.11	0.10	−0.09 – 0.31	1.09	0.277
	Time	−0.04	0.03	−0.11 – 0.03	−1.11	0.265
	A+/T−/N−	0.07	0.13	−0.18–0.32	0.53	0.599
	A+/T+/N+	1.34	0.13	1.09–1.59	10.47	**<0**.**0001**
	A+/T−/N−*Time	0.006	0.04	−0.08 – 0.09	0.13	0.894
	A+/T+/N+*Time	0.04	0.05	−0.05 – 0.13	0.89	0.374
	*Predictors*	*b*	*SE*	*95% CI*	*t*	*P*
**CSF Ng/BACE1**	Intercept	−0.78	0.11	−1.00 – −0.57	−7.11	**<0**.**0001**
	Age	−0.10	0.06	−0.22 – 0.02	−1.63	0.104
	APOE4 status	0.29	0.13	0.05–0.54	2.32	0.020
	Time	−0.10	0.05	−0.21–0.01	−1.86	0.063
	A+/T−/N−	0.56	0.15	0.26–0.87	3.65	0.**0003**
	A+/T+/N+	1.29	0.16	0.98–1.59	8.27	**<0**.**0001**
	A+/T−/N−*Time	0.03	0.07	−0.11 – 0.17	0.43	0.670
	A+/T+/N+*Time	0.14	0.08	−0.01 – 0.29	1.86	0.063
	*Predictors*	*b*	*SE*	*95% CI*	*t*	*P*
**CSF Aβ42/40**	Intercept	1.39	0.05	1.29–1.49	27.38	**<0**.**0001**
	Age	−0.05	0.03	−0.11 – 0.001	−1.94	0.052
	APOE4 status	−0.17	0.06	−0.28 – −0.05	−2.89	0.**004**
	Time	−0.06	0.03	−0.11 – −0.01	−2.54	0.011
	A+/T−/N−	−1.69	0.07	−1.83 – −1.55	−23.72	**<0**.**0001**
	A+/T+/N+	−2.02	0.07	−2.16 – −1.88	−28.10	**<0**.**0001**
	A+/T−/N−*Time	−0.01	0.03	−0.08 – 0.05	−0.42	0.673
	A+/T+/N+*Time	−0.002	0.03	−0.07 – 0.07	−0.07	0.946
	*Predictors*	*b*	*SE*	*95% CI*	*t*	*P*
**CSF p-tau**	Intercept	−0.74	0.07	−0.88 – −0.61	−10.91	**<0**.**0001**
	Age	0.10	0.04	0.02–0.17	2.52	0.012
	APOE4 status	0.07	0.08	−0.09 – 0.22	0.86	0.393
	Time	0.05	0.03	−0.02 – 0.11	1.35	0.178
	A+/T−/N−	0.30	0.10	0.12–0.49	3.19	0.**001**
	A+/T+/N+	1.80	0.10	1.61–1.99	18.69	**<0**.**0001**
	A+/T−/N−*Time	0.11	0.04	0.03–0.20	2.56	0.**01**
	A+/T+/N+*Time	0.02	0.05	−0.07 – 0.11	0.36	0.721
	*Predictors*	*b*	*SE*	*95% CI*	*t*	*P*
**CSF t−tau**	Intercept	−0.81	0.06	−0.94 – 0.69	−12.94	**<0**.**0001**
	Age	0.13	0.03	0.01–0.20	3.65	0.**0003**
	APOE4 status	0.07	0.07	−0.02 – 0.21	1.04	0.299
	Time	0.05	0.03	−0.01 – 0.10	1.62	0.105
	A+/T−/N−	0.42	0.09	0.05–0.59	4.75	**<0**.**0001**
	A+/T+/N+	1.86	0.09	0.97–2.03	20.91	**<0**.**0001**
	A+/T−/N−*Time	0.11	0.04	0.03–0.18	2.87	0.**004**
	A+/T+/N+*Time	−0.01	0.04	−0.04 – 0.06	−0.34	0.736

b, unstandardized regression coefficient; SE, standard error; CI, confidence interval; t, *t*-test statistic; *P*, *P*-value. Statistically significant results are highlighted in bold.

### Longitudinal trajectories of CSF markers in A/T/N stage progressors and non-progressors

**Figure 3:**
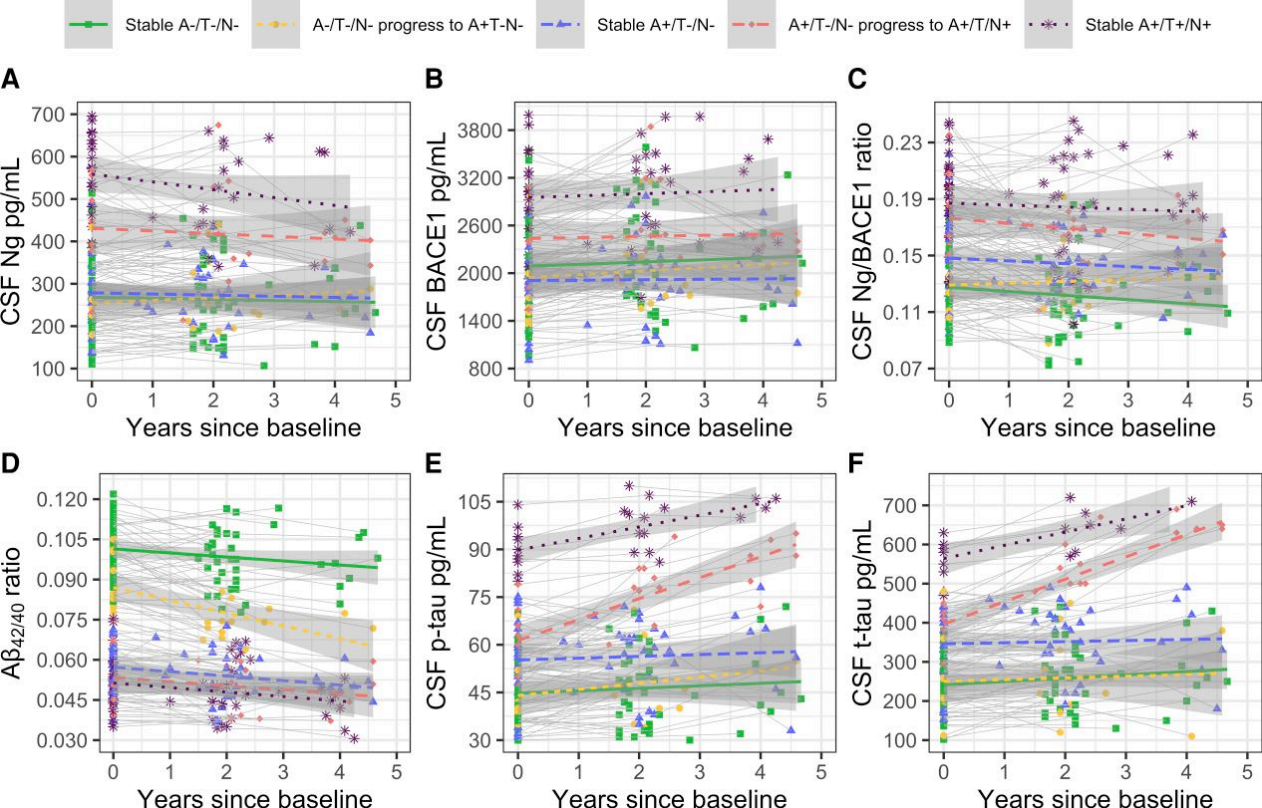
**Longitudinal change within and between A/T/N groups either remaining stable over time or progressing between A/T/N stages using LMMs. A–C** show longitudinal changes in Ng, BACE1 and Ng/BACE1 ratios. **D–F** show longitudinal changes in Aß_42/40_ ratios, p-tau and t-tau. All compared to stable A−/T−/N− controls: Ng only higher in A+/T−/N− progress to A+/T/N+ (*P* < 0.0001) and stable A+/T+/N+. BACE1 levels borderline significantly lower (*P* = 0.017) in stable A+/T−/N− and higher only in A+/T+/N+ (*P* < 0.0001). Ng/BACE1 ratios only higher in stable A+/T−/N− (*P* = 0.0004), A+/T−/N− progress to A+/T/N+ (*P* < 0.0001) and stable A+/T+/N+ (*P* < 0.0001). Ng, BACE1 and Ng/BACE1 remained stable over time in their respective A/T/N groups. Stable A+/T−/N− with slightly higher baseline t-tau levels (*P* = 0.002) that did not change over time (n.s.). An increase over time in t-tau (*P* < 0.0001) and p-tau (*P* < 0.0001) only evident for A+/T−/N− progress to A+/T/N+.

Please see Fig. 3, Table 3 (CSF synapse markers), and Table 4 (AD markers) for details. Cases that remained A+/T−/N− throughout the follow-up period had unaltered Ng levels (n.s.), at the threshold of significance (*P* = 0.017) lower BACE1 levels, and higher Ng/BACE1 levels (*P* = 0.0004) compared to controls. While not reaching diagnostic thresholds, this group had higher t-tau (*P* = 0.002) levels which did not increase over time. The increase in tau markers over time detailed in the main analyses was thus only evident in the subgroup of cases progressing from A+/T−/N− to A+/T/N+. This group also had higher Ng levels (*P* < 0.0001) and while not statistically significant, showed slightly higher BACE1 levels and higher Ng/BACE1 ratios (*P* < 0.0001), but none of these markers increased over time. For the stable A+/T+/N+ cases, Ng (*P* < 0.0001), BACE1 (*P* < 0.0001) and Ng/BACE1 (*P* < 0.0001) were at the highest levels among the groups and did not show significant increases over time. Interestingly, cases that progressed from A−/T−/N− to A+/T−/N− did not show significantly altered BACE1 or Ng levels over time. While we observed the expected decline in Aβ_42/40_ ratio for these progressors (*P* < 0.0001), these cases also had lower Aβ_42/40_ at baseline as compared to the stable A−T−N− cases (*P* < 0.0001).

**Table 3:** Longitudinal mixed linear models of CSF synapse marker change in A/T/N subgroups

	Predictors	b	SE	95% CI	T	*P*
**CSF BACE1**	Intercept	−0.16	0.14	−0.43 – 0.11	−1.18	0.238
	Age	0.24	0.08	0.09–0.39	3.07	0.**002**
	APOE	−0.09	0.15	−0.38 – 0.19	−0.63	0.528
	Time	0.05	0.04	−0.03 – 0.13	1.34	0.182
	A−/T−/N− to A+/T−/N−	−0.16	0.24	−0.63 – 0.31	−0.68	0.497
	Stable A+/T−/N−	−0.50	0.21	−0.91 – −0.09	−2.39	0.017
	A+/T−/N− to A+/T/N+	0.30	0.26	−0.22–0.81	1.13	0.259
	Stable A+/T+/N+	1.20	0.20	0.81–1.60	5.98	**<0**.**0001**
	A−/T−/N− to A+/T−/N−*Time	0.07	0.07	−0.07 – 0.21	0.98	0.328
	Stable A+/T−/N−*Time	−0.07	0.06	−0.19 – 0.04	−1.20	0.228
	A+/T−/N− to A+/T/N+*Time	-0.07	0.06	−0.20 – 0.06	−1.09	0.277
	Stable A+/T+/N+*Time	-0.10	0.05	−0.21 – 0.003	−1.90	0.057
**CSF Ng**	Intercept	−0.55	0.12	−0.78 – −0.31	−4.53	**<0**.**0001**
	Age	0.11	0.07	−0.02 – 0.24	1.60	0.110
	APOE	−0.03	0.13	−0.28 – 0.22	−0.22	0.825
	Time	−0.03	0.04	−0.10 – 0.05	−0.71	0.479
	A−/T−/N− to A+/T−/N−	0.02	0.21	−0.38 – 0.43	0.12	0.907
	Stable A+/T−/N−	0.03	0.18	−0.32 – 0.39	0.18	0.853
	A+/T−/N− to A+/T/N+	0.89	0.23	0.44–1.33	3.88	**<0**.**0001**
	Stable A+/T+/N+	1.72	0.18	1.38–2.07	9.83	**<0**.**0001**
	A−/T−/N− to A+/T−/N−*Time	0.14	0.07	0.01–0.26	2.06	0.038
	Stable A+/T−/N−*Time	0.01	0.05	−0.10–0.11	0.11	0.911
	A+/T−/N− to A+/T/N+*Time	-0.05	0.06	−0.17–0.07	−0.77	0.439
	Stable A+/T+/N+*Time	0.01	0.05	−0.09 – 0.11	0.22	0.823
**CSF Ng/BACE1**	Intercept	−0.84	0.13	−1.09– −0.59	−6.62	**<0**.**0001**
	Age	−0.12	0.07	−0.26 – 0.02	−1.75	0.081
	APOE	0.09	0.13	−0.17 – 0.36	0.70	0.481
	Time	−0.10	0.07	−0.23 – 0.04	−1.38	0.168
	A−/T−/N− to A+/T−/N−	0.23	0.22	−0.20 – 0.66	1.03	0.302
	Stable A+/T−/N−	0.68	0.19	0.31–1.06	3.56	0.**0004**
	A+/T−/N− to A+/T/N+	1.27	0.24	0.80–1.74	5.27	**<0**.**0001**
	Stable A+/T+/N+	1.77	0.19	1.41–2.13	9.55	**<0**.**0001**
	A−/T−/N− to A+/T−/N−*Time	0.17	0.12	−0.07 – 0.41	1.38	0.169
	Stable A+/T−/N−*Time	0.06	0.10	−0.15 – 0.26	0.55	0.583
	A+/T−/N− to A+/T/N+*Time	-0.05	0.11	−0.27 – 0.18	−0.42	0.677
	Stable A+/T+/N+*Time	0.17	0.10	−0.02 – 0.36	1.79	0.074

b, unstandardized regression coefficient; SE, standard error; CI, confidence interval; t, *t*-test statistic; *P*, *P*-value. Statistically significant results are highlighted in bold.

**Table 4:** Longitudinal mixed linear models of CSF Alzheimer’s disease marker change in A/T/N subgroups

	*Predictors*	*b*	*SE*	*95% CI*	*T*	*P*
**CSF Aβ42/40**	Intercept	1.26	0.07	1.12–1.40	17.37	**<0**.**0001**
	Age	−0.11	0.04	−0.19 – −0.03	−2.71	0.**007**
	APOE	−0.12	0.08	−0.27 – 0.03	−1.52	0.128
	Time	−0.07	0.03	−0.13 – −0.01	−2.13	<0.05
	A−/T−/N− to A+/T−/N−	−0.76	0.13	−1.01 – −0.52	−6.09	**<0**.**0001**
	Stable A+/T−/N−	−1.69	0.11	−1.91 – −1.48	−15.45	**<0**.**0001**
	A+/T−/N− to A+/T/N+	−1.86	0.14	−2.13 – −1.59	−13.55	**<0**.**0001**
	Stable A+/T+/N+	−1.98	0.11	−2.19 – −1.77	−18.73	**<0**.**0001**
	A−/T−/N− to A+/T−/N−*Time	−0.26	0.05	−0.37 – −0.16	−4.82	**<0**.**0001**
	Stable A+/T−/N−*Time	−0.01	0.05	−0.10 – 0.08	−0.20	0.841
	A+/T−/N− to A+/T/N+*Time	-0.02	0.05	−0.12– 0.08	−0.37	0.711
	Stable A+/T+/N+*Time	-0.01	0.04	−0.09–0.07	−0.21	0.834
**CSF p-tau**	Intercept	−0.66	0.09	−0.84 – −0.48	−7.38	**<0**.**0001**
	Age	0.13	0.05	0.03–0.23	2.62	<0.05
	APOE	−0.01	0.09	−0.19–0.18	−0.10	0.919
	Time	0.07	0.04	−0.01–0.15	1.79	0.073
	A−/T−/N− to A+/T−/N−	−0.08	0.16	−0.38–0.23	−0.50	0.614
	Stable A+/T−/N−	0.31	0.14	−0.04–0.57	2.28	0.023
	A+/T−/N− to A+/T/N+	0.89	0.17	0.56–1.23	5.26	**<0**.**0001**
	Stable A+/T+/N+	1.95	0.13	1.69–2.20	14.90	**<0**.**0001**
	A−/T−/N− to A+/T−/N−*Time	−0.03	0.07	−0.17–0.11	−0.40	0.689
	Stable A+/T−/N−*Time	0.001	0.06	−0.12–0.12	−0.02	0.981
	A+/T−/N− to A+/T/N+*Time	0.21	0.07	0.08–0.35	3.22	0.**0001**
	Stable A+/T+/N+*Time	-0.02	0.06	−0.13–0.09	−0.31	0.755
**CSF t-tau**	Intercept	−0.72	0.09	−0.90– −0.55	−8.13	**<0**.**0001**
	Age	0.14	0.05	0.04–0.23	2.71	0.**007**
	APOE	−0.001	0.09	−0.19–0.18	−0.01	0.990
	Time	0.07	0.04	−0.001–0.14	1.95	0.052
	A−/T−/N− to A+/T−/N−	−0.01	0.15	−0.32–0.29	−0.09	0.932
	Stable A+/T−/N−	0.42	0.13	−0.16–0.69	3.13	0.**002**
	A+/T−/N− to A+/T/N+	1.01	0.17	0.68–1.35	6.01	**<0**.**0001**
	Stable A+/T+/N+	1.99	0.13	1.73–2.24	15.30	**<0**.**0001**
	A−/T−/N− to A+/T−/N−*Time	0.02	0.09	−0.10–0.15	0.36	0.722
	Stable A+/T−/N−*Time	−0.01	0.05	−0.12–0.09	−0.25	0.806
	A+/T−/N− to A+/T/N+*Time	0.23	0.06	0.12–0.35	3.97	**<0**.**0001**
	Stable A+/T+/N+*Time	−0.04	0.05	−0.14–0.06	−0.82	0.411

b, unstandardized regression coefficient; SE, standard error; CI, confidence interval; t, *t*-test statistic; *P*, *P*-value. Statistically significant results are highlighted in bold.

### Longitudinal relationships between CSF synaptic biomarkers and memory decline

**Figure 4:**
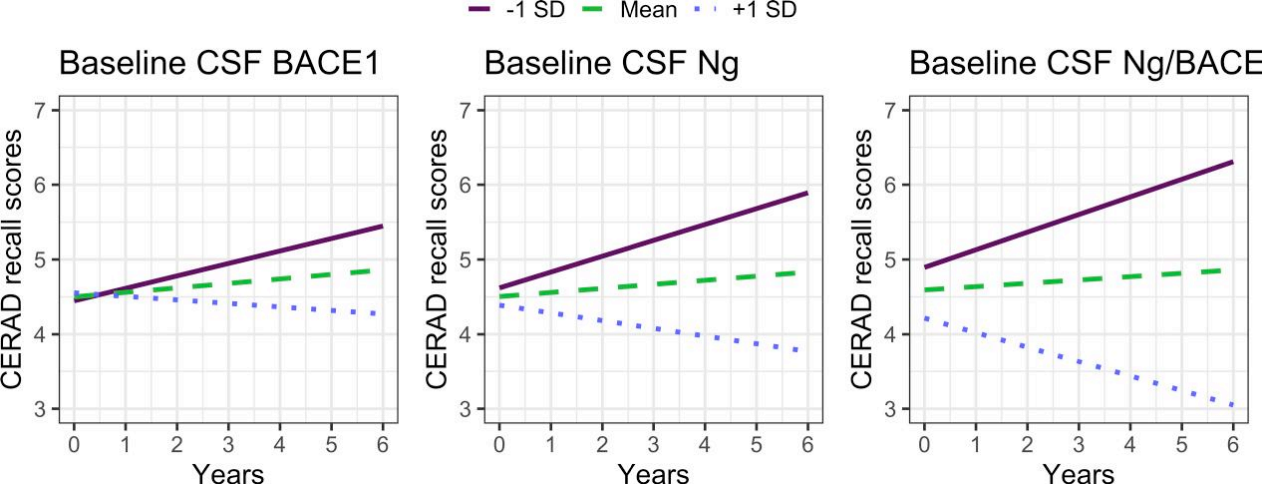
**CSF synapse marker associations with CERAD delayed memory decline.** Ng (*b* = −0.20, *P* = 0.002), BACE1 (*b* = −0.13, *P* = 0.046), Ng/BACE1 ratio (*b* = −0.29, *P* = 0.0006) by time interaction effect on CERAD memory decline, respectively. Plots are produced with predicted values from the longitudinal LMMs. The lines represent the effect on CERAD memory decline over time when the baseline biomarker levels are at the mean, or −1SD or +1SD below or above the mean.

Both baseline Ng (*b* = −0.20, *P* = 0.002) and Ng/BACE1 (*b* = 0.29, *P* = 0.0006) predicted CERAD memory decline over time. BACE1 did not reach the threshold for statistical significance (*b* = 0.13, *P* = 0.046). As illustrated in Fig. 4, these results suggest that Ng/BACE1 shows a better association with memory performance and decline than Ng. Detailed results from the longitudinal mixed linear models are shown in Table 5.

**Table 5:** Longitudinal mixed linear models detailing fixed effects estimates associated with CERAD delayed memory recall decline

	Predictors	b	SE	95% CI	t	*P*
**CSF BACE1**	Intercept	2.48	2.56	−2.54–7.50	0.97	0.333
	Age	−0.59	0.12	−0.82 – −0.36	−5.09	**<0**.**0001**
	Years of education	0.12	0.03	0.06–0.19	3.62	0.**0003**
	Sex (Female)	0.60	0.22	0.17–1.03	2.72	**<0**.**006**
	Time	0.08	0.08	−0.07–0.24	1.07	0.284
	CSF BACE1	−0.07	0.12	−0.30–0.17	−0.57	0.572
	Time*CSF BACE1	−0.13	0.07	−0.27 – −0.01	−2.00	0.046
**CSF Ng**	Intercept	2.49	2.54	−2.50–7.47	0.98	0.328
	Age	−0.56	0.11	−0.78 – −0.33	−4.89	**<0**.**0001**
	Years of education	0.12	0.03	0.06–0.19	3.65	0.**0003**
	Sex (Female)	0.61	0.22	0.18–1.03	2.79	0.**005**
	Time	0.08	0.08	−0.07–0.23	1.04	0.298
	CSF Ng	-0.25	0.11	−0.47 – −0.03	−2.19	0.028
	Time*CSF Ng	−0.20	0.07	−0.34 – −0.07	−3.02	0.**002**
**CSF Ng/BACE1**	Intercept	2.37	2.45	−2.44–7.18	0.96	0.502
	Age	-0.58	0.11	−0.80 – −0.37	−5.32	**<0**.**0001**
	Years of education	0.13	0.03	0.07–0.20	3.97	**<0**.**0001**
	Sex (Male)	0.70	0.21	0.28–1.11	3.27	0.**001**
	Time	0.04	0.08	−0.11–0.19	0.52	0.605
	CSF Ng/BACE1	−0.51	0.11	−0.72 – −0.29	−4.67	**<0**.**0001**
	Time*CSF Ng/BACE1	−0.29	0.08	−0.45 – −0.12	−3.48	0.**0006**

b, unstandardized regression coefficient; SE, standard error; CI, confidence interval; t, t−test statistic; *P*, *P*-value. Statistically significant results are highlighted in bold.

### Supplementary comparisons of Aß_1-42_ Aß_1-40_ and Aß_42/40_ ratio levels between baseline A/T/N groups

**Figure 5:**
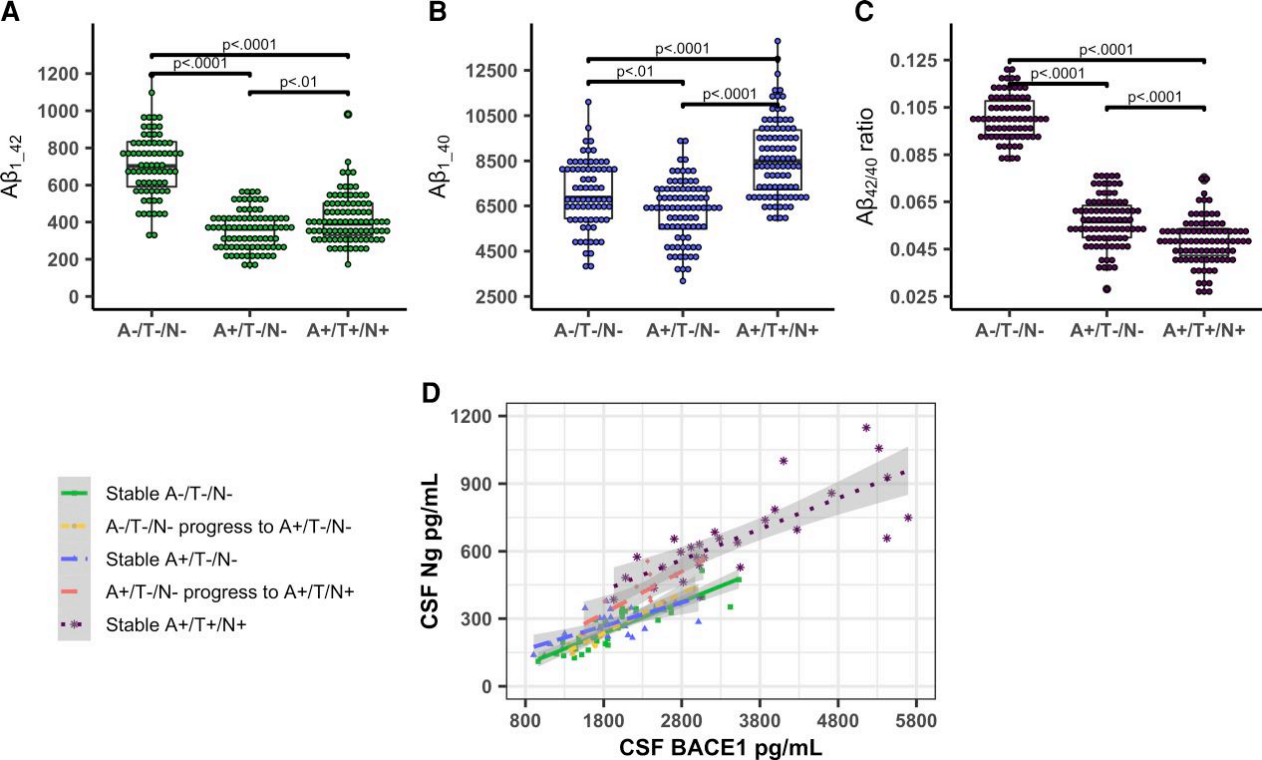
**Aß species in A/T/N groups and correlations between the Ng and BACE1.** Comparison of Aß_1-40_ levels (**A**) Aß_1-42_ (**B**) and Aß_42/40_ ratio (**C**) between A/T/N groups at baseline. The brackets mark the statistical comparisons of interest following a significant ANCOVA main-effect between the A/T/N groups. The *P*-values are in accordance with the threshold following the Bonferroni-Holm procedure. Illustrating Pearson’s product-moment correlations between baseline Ng and BACE1 in different A/T/N progressors and non-progressors (**D).** Stable A−/T−/N− (*r* = 0.888, *R^2^*=0.789, *P* < 0.001); A−/T−/N− progress to A+/T−/N− (*r* = 0.954, *R^2^*=0.909, *P* < 0.001); stable A+/T−/N− cases (*r* = 0.659, *R^2^* = 0.434, *P* < 0.001); A+/T−/N− progressed to A+/T/N+ (*r* = 0.772, *R^2^*=0.596, *P* < 0.003); stable A+/T+/N+ (*r* = 0.779, *R^2^* = 0.606, *P* < 0.001).

The A+/T+/N+ group had lower Aß_1-42_ and Aß_42/40_ ratio levels compared to A+/T−/N− (*P* < 0.01; *P* < 0.0001). Aß_1-40_ levels were lower in A+/T−/N− (*P* < 0.01) and higher in A+/T+/N+ (*P* < 0.0001) as compared to A−/T−/N− controls (see Figs. 5A–5C).

### Supplementary correlations of Ng and BACE1 in different A/T/N stage progressors and non-progressors

CSF Ng and BACE1 levels showed moderate to high correlations in all groups, but the highest correlations were shown in cases that were stable A−/T−/N− (*r* = 0.888, *R*^2^ = 0.789, *P* < 0.001) and A−/T−/N− that later progressed to A+/T−/N− (*r* = 0.954, *R*^2^ = 0.909, *P* < 0.001). Both A+/T−/N− that later progressed to A+/T/N+ and stable A+/T+/N+ showed slightly lower, albeit similar correlations (*r* = 0.772, *R^2^* = 0.596, *P* < 0.003 and *r* = 0.779, *R*^2^ = 0.606, *P* < 0.001 respectively). In contrast, the lowest, although still moderate correlation was found in stable A+/T−/N− cases (*r* = 0.659, *R*^2^ = 0.434, *P* < 0.001) (see Fig. 5D).

### Supplementary descriptive comparison of progression to dementia between stable A+/T−/N− and stable A+/T+/N+

Of note, a similar proportion of cases remaining stable A+/T−/N− (*n* = 5, 19.2%) and stable A+/T+/N+ (*n* = 5, 17.9%) progressed to dementia in the follow-up period (see Supplementary Fig. 1 for additional details).

## Discussion

Ng and BACE1 are both linked to attractive drug targets in Alzheimer’s disease, but treatment trials have failed to demonstrate positive effects on disease progression.^26,35^ While increased Ng and BACE1 levels are linked to Alzheimer’s disease pathology and symptoms, they are differentially expressed between patients. If expression levels are consistent across phenotypes, this may point to differential activation of pathological mechanisms between patients, implying a need for different treatment strategies. Here, we show that longitudinal measures of CSF Ng, BACE1, and Ng/BACE1 levels are consistent traits across the A/T/N stages of the Alzheimer’s disease continuum. At baseline, mean Ng and BACE1 levels were only slightly higher compared to A−/T−/N− healthy control cases at the A+/T+/N+ stage, whereas Ng was unaltered, and BACE1 was reduced at the A+/T−/N− stage. But, the Ng/BACE1 ratio was increased in both A+ groups. Within A/T/N− groups, longitudinal data showed stable Ng, BACE1 and Ng/BACE1 CSF levels over time. CSF tau levels were higher in the A+/T−/N− cases and increased over time. A sub-analysis of these groups showed that the A+/T−/N− cases consisted of both A+/T−/N− cases that remained A+/T−/N− over time and cases that progressed towards significant amounts of tau-pathology (i.e. converted to A+/T or N+). These progressors also had consistently higher Ng levels and, while not significant, numerically elevated BACE1 levels. While tau levels increased over time for the progressors, both groups had higher tau levels than the A−/T−/N− controls. In contrast, the stable A+/T−/N− cases, though showing sub-threshold CSF tau elevation, had numerically but not significantly lower BACE1 levels. However, the amyloid negative subjects (A−/T−/N−) who converted to an amyloid positive stage (A+/T−/N−) showed no significant alterations in BACE1 or Ng levels.

As described, synaptic structures are sensitive to Aß oligomers (see introduction),^9,10^ and recent studies point to pathological effects of Aß oligomers on both pre- and postsynaptic structures.^6,7,52,53^ BACE1 is predominately a pre-synaptic enzyme associated with synaptic vesicles and is known to have several synaptic proteins as substrates, including neuregulin and seizure protein 6, which are important for myelination and synaptic plasticity.^11,54^ Indeed, BACE1 inhibition has been shown to produce cognitive deficits in both animal models and human trials.^11,28^ Thus, reduced BACE1 levels in A+/T−/N− cases may reflect altered processing of synaptic substrates dependent on BACE1 cleavage. While these cases had Aβ_42/40_ ratios consistent with amyloid plaque deposition, they also had lower CSF Aβ_1-42_ and Aβ_1-40_ concentrations as compared to both controls and A+/T+/N+ cases (Figs. 5A–C). As described, experimental data suggests that Aβ oligomers may have a reciprocal stimulatory effect on pre-synaptic BACE1 activity, and both oligomers, and APP fragments including Aβ species regulate synaptic transmission.^7,55^ These mechanisms are incompletely understood, but reductions in Aβ species in A+/T−/N− cases could be related to presynaptic dysfunction, reduced synaptic activity and lowered levels of CSF BACE1.^56^ Conversely, the markedly higher BACE1 levels shown in the A+/T+/N+ group may result from synapse degradation and BACE1 release. Alternatively, glial activation leading to increased BACE1 expression in reactive astrocytes could correspond to the increased Aβ_1-40_ levels in this group (Fig. 5B).^57,58^ Reactive glia (astrocytes and microglia) may lose their function in synaptic homeostasis and instead contribute to inflammation, and putatively to neurotoxicity and further neurodegeneration.^59^ We have previously shown (in a subset of the same cases) that astrocyte activation markers such as clusterin and chitinase-3-like protein 1 (YKL-40) were increased in cases with both amyloid pathology and tau pathology (A+/T+/N+), but not in cases with amyloid pathology only (A+/T−/N).^60^ Higher Ng levels in A+/T+/N+ cases are consistent with degenerative and inflammatory pathology with Ng-release to the interstitial fluid.^14^ However, the closely correlated BACE1 and Ng levels (Fig. 5D) suggest that synapse-related processes may be the major drivers of release to the CSF for both markers and that the increased CSF levels mainly reflect synaptic pathologies (though increased astroglial BACE1 expression may also be involved). Moreover, the strength of the Ng and BACE1 correlations varied considerably between the A/T/N groups. Importantly, all groups with pathological Alzheimer’s disease biomarkers show lower effect sizes (between 43 and 61% shared variance) as compared to those with normal biomarkers (between 79 and 91% shared variance). Of note, the pattern of differently altered BACE1 and Ng levels in A+/T−/N− and A+/T+/N+ groups resulted in a stage-wise elevation of Ng/BACE1 ratios through the Alzheimer’s disease continuum and was more strongly associated with baseline memory performance and later decline than Ng or BACE1 alone. Thus, the altered relationships between Ng and BACE1 in both A+ groups could reflect compromised pre- and postsynaptic integrity when expressed as a ratio.^17^ In summary, the differential expression of BACE1 and Ng along the Alzheimer’s disease continuum may relate to pathological changes at both pre-and post-synaptic terminals and is consistent with synapse degradation and altered neuron-glia interactions at more advanced stages.

Elevated CSF tau in A+/T−/N− cases even at subthreshold levels, is consistent with recent findings pointing to Aβ induced tau pathology and a gradual transition towards more advanced stages along the traditional A/T/N trajectory as a major pathway.^2,61^ However, we found that CSF levels of Ng, BACE1 and Ng/BACE1 remained largely stable within the strata for the A/T/N groups over time. Surprisingly, this was also the case for those progressing from A+/T−/N− to A+/T or N+. Moreover, we did not observe an initial reduction in BACE1 levels at the onset of amyloid plaque formation or an increase over time of BACE1 and Ng in neither stable A+/T−/N− cases nor A+/T−/N− cases that progressed to A+/T or N+. However, we found consistently higher Ng levels in cases transitioning from A+/T−/N− to A+/T or N+. Thus, longitudinal data does not show BACE1, Ng or Ng/BACE1 changes coinciding with stage-wise Alzheimer’s disease progression but gives evidence of lasting differences between cases over the observation period, also with increasing core pathologies. For example, a high Ng level and a Ng/BACE1 ratio at baseline in future A+/T−/N− to A+/T/N+ progressors may indicate a latent phenotype where pathological mechanisms for progression are increasingly active. This interpretation is also supported by the high predictive value of Ng and Ng/BACE1 for deteriorating CERAD recall scores (Fig. 4).

Recent studies have outlined putative mechanistic subtypes with distinct biomarker profiles linked to genetic variance.^4,5^ Both glial and innate immune activation as well as synaptic pathology, could contribute to subgroup definition, as expressed by CSF levels of proteins such as Ng, BACE1, t- and p-tau. This is partly consistent with our findings, where Ng and BACE1 levels are stable within A/T/N groups and there are indications of numerically lower BACE1 in stable A+/T−/N− cases over time as compared to controls (i.e. *P* < 0.05, above the specified α-level at 0.01). A high fraction (*n* = 26, 39.4% at the second visit, see Supplementary Table 2) of predementia A+/T−/N− cases included in our study had stable sub-threshold CSF tau levels over time. However, *n* = 5 (19.2%) of stable A+/T−/N− cases and *n* = 5 (17.9%) of stable A+/T+/N+ cases were diagnosed with dementia during the follow-up period (supplementary Fig. 1). Though these numbers are relatively small, the finding is in line with a recent longitudinal study where 45% of clinically diagnosed Alzheimer’s disease dementia cases did not show pathological levels of CSF p-tau or t-tau, and did not differ in cognitive or functional decline as compared to cases with pathological tau levels (i.e. A+/T+/N+).^62^ Moreover, it has been shown that up to 30% of autopsy-confirmed Alzheimer’s disease patients have normal CSF t-tau levels.^63^

These cases were followed and had repeated CSF examinations over a timeframe relevant for drug interventions and showed stable characteristics in terms of markers for ongoing synapse pathology during this period. Thus, these findings support using stratification for synapse pathology to focus new trials with protective substances such as NMDA-blockers in cases with evidence of high levels of synapse degeneration. As discussed, trials with BACE1 inhibitors have not met endpoints, but novel approaches employing low-level inhibition have not been explored, nor has stratification according to levels of synapse pathology. The apparently concordant increase of Ng and BACE1 in A+/T+/N+ cases (Figs. 1, 2 and 3) could support use of both pre-and post-synaptic agents in these cases, e.g. in the form of BACE1 inhibitors and NMDA blockers.

This study has some limitations. We plan to extend the set of synapse markers analyzed to cover more aspects of pre- and post-synaptic signalling and pathologies. BACE1 and Ng patient differences could reflect genetic susceptibilities for synapse pathologies, which will be explored in future work. CSF total tau correlates strongly with p-tau, and the use of total-tau as a general marker of neurodegeneration (N) may obscure cases with non-tau mediated neurodegeneration reflected by markers such as neurofilament light chain (NFL). However, this marker was not available in the DDI cohort at the time of analysis. Moreover, the sub-analysis of cases transitioning between or remaining within their A/T/N stage was limited by a low number of cases. Results must therefore be interpreted with some caution. In addition, an important limitation in this study is the exclusion of cognitive status in the A+ groups (i.e. cognitively normal with or without SCD or MCI) in our analyses, as this would have further reduced our statistical power. However, we sought to adjust for this shortcoming by including cognitive status as a random effect in models associating CSF markers with memory recall performance and decline. Nevertheless, if levels of Ng and BACE1 reflect relevant synapse pathologies within the AD-continuum, we should expect marker levels to also differ along the clinical continuum of AD. This should also hold true between, and within, putative pathomechanistic subgroups and along the clinical continuum towards dementia. The DDI study is continuously following up included cases and we plan to carry out such a study when we have enough data for longitudinal statistical analysis.

## Conclusions

Ng, BACE1 and Ng/BACE1 CSF levels are consistent phenotypes across A/T/N-stages and may present different Alzheimer’s disease subgroups rather than reflecting disease progression. The Ng/BACE1 ratio is a predictor for reduced cognition throughout the Alzheimer’s disease continuum and may serve as a biomarker for synaptic dysfunction. These differences may point to underlying pathomechanistic factors that may allow for different treatment options.

## Supplementary Material

fcac244_Supplementary_Data

## Abbreviations

A+/− =: positive or negative Cerebrospinal fluid (CSF) marker for amyloid plaques
Aβ =: amyloid-beta
ANCOVA =: analysis of covariance
APOE =: apolipoprotein
APP =: amyloid precursor protein
BACE1 =: β-site APP cleaving enzyme 1
CERAD =: consortium to establish a registry for Alzheimer’s disease
LMM =: linear mixed models
MCI =: mild cognitive impairment
N+/− =: positive or negative marker for neurodegeneration
NMDA =: N-methyl-D-aspartate
Ng =: neurogranin
SCD =: subjective cognitive decline
T+/- =: positive or negative marker for tau-tangle pathology
