# Supplementary material for: Stable cerebrospinal fluid neurogranin and β-site amyloid precursor protein cleaving enzyme 1 levels differentiate predementia Alzheimer’s disease patients
Source: Brain Commun. 2022 Sep 24;4(5):fcac244. doi: 10.1093/braincomms/fcac244 (PMC9562788; doi:10.1093/braincomms/fcac244)
Supplement: fcac244_Supplementary_Data [file fcac244_supplementary_data.docx]

| **Supplementary table 1.** Sample descriptives of main A/T/N groups with repeated CSF samples. | | | | |
| --- | --- | --- | --- | --- |
|  |  | A-/T-/N- | A+/T-/N- | A+/T+/N+ |
| Baseline | n | 74 | 86 | 90 |
|  | Years since baseline Mean (SD) | ^a^ | ^a^ | ^a^ |
|  | Age Mean (SD) | 60.9 (8.3) | 67.9 (7.4) | 68.0 (8.4) |
|  | Female n (%) | 44 (59.5) | 52 (60.5) | 46 (51.1) |
|  | *APOE-ε4+* n (%) | 28 (37.8) | 65 (75.6) | 71 (78.9) |
|  |  |  |  |  |
| First visit | n | 33 | 39 | 28 |
|  | Years since baseline Mean (SD) | 1.99 (0.31) | 2.02 (0.61) | 2.02 (0.37) |
|  | Age Mean (SD) | 61.9 (8.33) | 71.1 (7.3) | 70.3 (7.4) |
|  | Female n (%) | 18 (54.5) | 21 (53.8) | 12 (42.9) |
|  | *APOE-ε4+* n (%) | 12 (36.4) | 27 (69.2) | 18 (64.3) |
|  |  |  |  |  |
| Second visit | n | 8 | 11 | 10 |
|  | Years since baseline Mean (SD) | 4.14 (0.32) | 4.18 (0.35) | 4.11 (0.63) |
|  | Age Mean (SD) | 64.1 (7.5) | 74.4 (7.5) | 75.4 (4.1) |
|  | Female n (%) | 6 (75) | 6 (54.5) | 5 (50.0) |
|  | *APOE-ε4+* n (%) | 1 (12.5) | 8 (72.7) | 6 (60.0) |
| Abbreviations: A+/-, positive or negative CSF marker for amyloid plaques; N+/-, positive or negative marker for neurodegeneration; SD, standard deviation; n, number of cases; %, percentage; ^a^, no value. No between-group comparisons performed. | | | | |

| **Supplementary table 2.** Sample descriptives of sub-sample A/T/N groups with repeated CSF samples. | | | | | | |
| --- | --- | --- | --- | --- | --- | --- |
| Visit | Variables | Stable  A-/T-/N- | A-/T-/N- progress to  A+/T-/N- | Stable  A+/T-/N- | A+/T-/N- progress to A+/T/N+ | Stable  A+/T+/N+ |
| Baseline | n | 33 | 12 | 26 | 12 | 28 |
|  | Years since baseline Mean (SD) | ^a^ | ^a^ | ^a^ | ^a^ | ^a^ |
|  | Age Mean (SD) | 59.8 (8.3) | 62.9 (9.6) | 69.5 (7.0) | 67.3 (8.1) | 68.1 (7.5) |
|  | Female n (%) | 18 (54.5) | 5 (41.7) | 14 (53.8) | 6 (50.0) | 12 (42.9) |
|  | *APOE-ε4+* n (%) | 12 (36.4) | 6 (50.0) | 16 (61.5) | 11 (91.7) | 18 (64.3) |
|  |  |  |  |  |  |  |
| First visit | n | 33 | 12 | 26 | 12 | 28 |
|  | Years since baseline Mean (SD) | 2.0 (0.3) | 2.0 (0.4) | 1.9 (0.5) | 2.4 (0.8) | 2.0 (0.4) |
|  | Age Mean (SD) | 61.9 (8.3) | 65.3 (9.6) | 71.4 (7.1) | 69.6 (68.5) | 70.3 (7.4) |
|  | Female n (%) | 18 (54.5) | 5 (41.7) | 14 (53.8) | 6 (50.0) | 12 (42.9) |
|  | *APOE-ε4+* n (%) | 12 (36.4) | 6 (50.0) | 16 (61.5) | 11 (91.7) | 18 (64.3) |
|  |  |  |  |  |  |  |
| Second visit | n | 8 | 3 | 7 | 4 | 10 |
|  | Years since baseline Mean (SD) | 4.1 (0.3) | 4.7 (0.6) | 4.1 (0.4) | 4.3 (0.4) | 4.1 (0.6) |
|  | Age Mean (SD) | 64.1 (7.5) | 62.0 (8.9) | 74.9 (8.7) | 73.5 (5.7) | 75.4 (4.1) |
|  | Female n (%) | 6 (75) | 2 (66.7) | 3 (42.9) | 3 (75.0) | 5 (50.0) |
|  | *APOE-ε4+* n (%) | 1 (12.5) | 2 (66.7) | 4 (57.1) | 4 (100.0) | 6 (60.0) |
| Abbreviations: A+/-, positive or negative CSF marker for amyloid plaques; N+/-, positive or negative marker for neurodegeneration; SD, standard deviation; n, number of cases; %, percentage; ^a^, no value. No between group comparisons performed. | | | | | | |


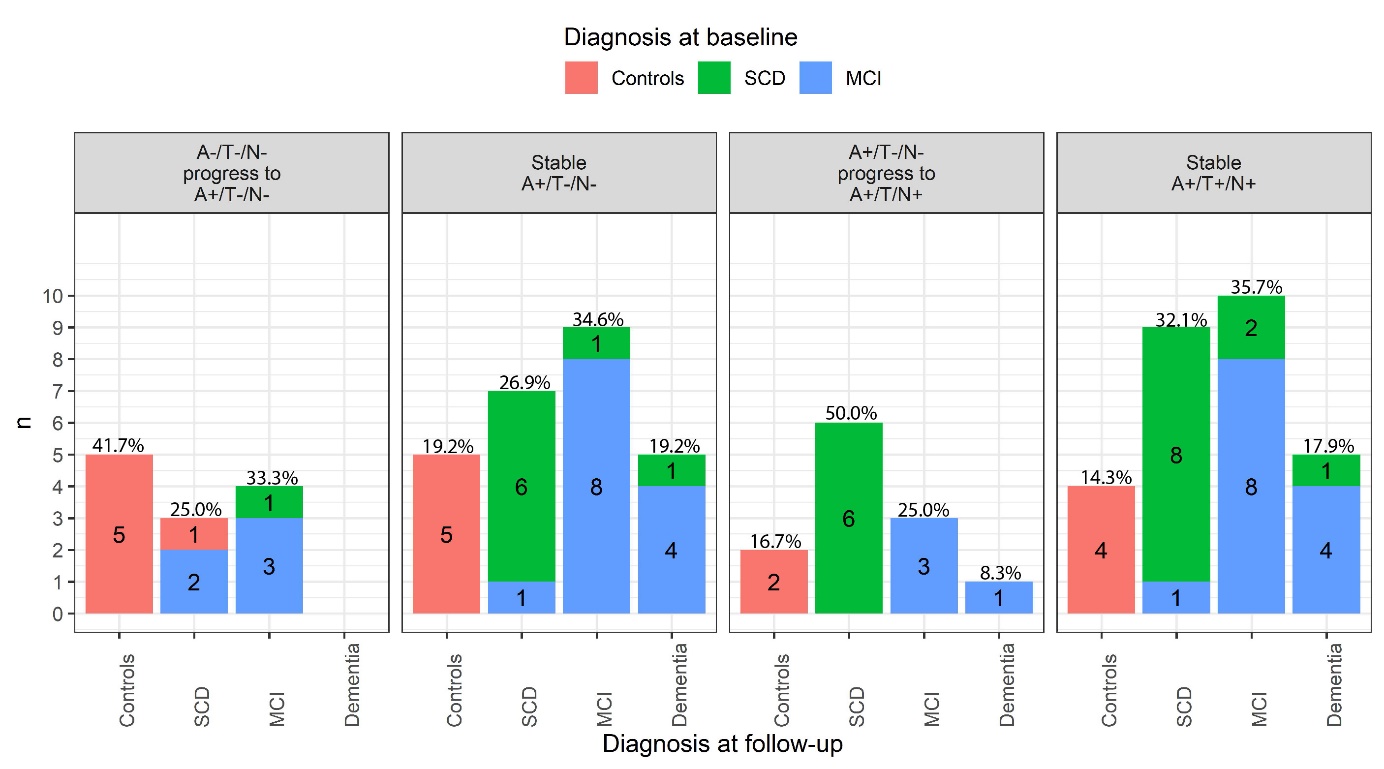


**Supplementary figure 1.** A stacked bar chart showing number (n) and percentage (%) of cases changing or retaining baseline diagnosis over time within respective A/T/N groups. Please note that the follow-up diagnosis is noted at the x-axis, while the baseline diagnosis is indicated by colors (legend).
